# Insufficient Fruit and Vegetable Intake and Low Potassium Intake Aggravate Early Renal Damage in Children: A Longitudinal Study

**DOI:** 10.3390/nu14061228

**Published:** 2022-03-14

**Authors:** Menglong Li, Nubiya Amaerjiang, Ziang Li, Huidi Xiao, Jiawulan Zunong, Lifang Gao, Sten H. Vermund, Yifei Hu

**Affiliations:** 1 Department of Child, Adolescent Health and Maternal Care, School of Public Health, Capital Medical University, Beijing 100069, China; limenglong_ph@163.com (M.L.); 13693617970@163.com (N.A.); 15622764530@163.com (Z.L.); xhd19988023@163.com (H.X.); jiawulan@foxmail.com (J.Z.); lifanggao@ccmu.edu.cn (L.G.); 2 Yale School of Public Health, Yale University, New Haven, CT 06510-3201, USA; sten.vermund@yale.edu

**Keywords:** fruit and vegetable intake, potassium intake, renal damage, children, China

## Abstract

Insufficient fruit and vegetable intake (FVI) and low potassium intake are associated with many non-communicable diseases, but the association with early renal damage in children is uncertain. We aimed to identify the associations of early renal damage with insufficient FVI and daily potassium intake in a general pediatric population. We conducted four waves of urine assays based on our child cohort (PROC) study from October 2018 to November 2019 in Beijing, China. We investigated FVI and other lifestyle status via questionnaire surveys and measured urinary potassium, β_2_-microglobulin (β_2_-MG), and microalbumin (MA) excretion to assess daily potassium intake and renal damage among 1914 primary school children. The prevalence of insufficient FVI (<4/d) was 48.6% (95% CI: 46.4%, 50.9%) and the estimated potassium intake at baseline was 1.63 ± 0.48 g/d. Short sleep duration, long screen time, lower estimated potassium intake, higher β_2_-MG and MA excretion were significantly more frequent in the insufficient FVI group. We generated linear mixed effects models and observed the bivariate associations of urinary β_2_-MG and MA excretion with insufficient FVI (*β* = 0.012, 95% CI: 0.005, 0.020; *β* = 0.717, 95% CI: 0.075, 1.359), and estimated potassium intake (*β* = −0.042, 95% CI: −0.052, −0.033; *β* = −1.778, 95% CI: −2.600, −0.956), respectively; after adjusting for age, sex, BMI, SBP, sleep duration, screen time and physical activity. In multivariate models, we observed that urinary β_2_-MG excretion increased with insufficient FVI (*β* = 0.011, 95% CI: 0.004, 0.018) and insufficient potassium intake (<1.5 g/d) (*β* = 0.031, 95% CI: 0.023, 0.038); and urinary MA excretion increased with insufficient FVI (*β* = 0.658, 95% CI: 0.017, 1.299) and insufficient potassium intake (*β* = 1.185, 95% CI: 0.492, 1.878). We visualized different quartiles of potassium intake showing different renal damage with insufficient FVI for interpretation and validation of the findings. Insufficient FVI and low potassium intake aggravate early renal damage in children and underscores that healthy lifestyles, especially adequate FVI, should be advocated.

## 1. Introduction

Insufficient fruit and vegetable intake (FVI) is associated with almost all major non-communicable diseases [1], including emerging chronic kidney disease (CKD) in the general population [2]. Adequate FVI plays an important role in ensuring good development in children and adolescents, reducing risk of obesity and micronutrient deficiencies and improving health status in adulthood [3,4]. The unmet recommended standard of FVI in terms of composition and frequency among children ages 6–17 years in China has gained attention in efforts to optimize childhood diets [5,6].

Dietary intake affects the acid-base balance in the human body. Plant-based foods including fruits and vegetables are rich in mineral cations (such as potassium [7]) and bicarbonate precursors with alkalizing effects [8]. Insufficient FVI is associated with increased acidity and kidney function declines in CKD patients [8]. High consumption of fruits and vegetables may have a buffering effect on metabolic acid load [9]. This is postulated as having a potentially protective role in avoiding renal damage. One study showed that increased FVI intake for 8 weeks was associated with a decrease in net endogenous acid production (NEAP) [10].

Potassium is a major factor in NEAP, and estimated NEAP is negatively correlated with potassium intake [8]. Studies among adults have shown that higher dietary acid load is associated with CKD, and higher intake of potassium is negatively associated with CKD [11]. Potassium is freely filtered at the glomerulus, with approximately 60–80% of the filtered potassium reabsorbed in the proximal tubule and 10% excreted in the distal tubule [12]. Increased β_2_-microglobulin (β_2_-MG) and microalbumin (MA) in the urine are markers of early renal damage [13,14], and are used to assess a worse status of renal proximal tubular and glomerular function, respectively [15]. A prospective, population-based cohort study conducted in adults with normal renal function showed that low urinary potassium excretion was associated with an increased risk of CKD [16] and another young adult study suggested that higher potassium intake may help prevent renal damage [17].

The effects of FVI and potassium intake on renal damage have not been studied among school-aged healthy children, and we sought to determine their associations using longitudinal data. We hypothesized that insufficient FVI and lower potassium intake may aggravate early renal damage in children.

## 2. Materials and Methods

### 2.1. Study Design and Participants

**Figure 1:**
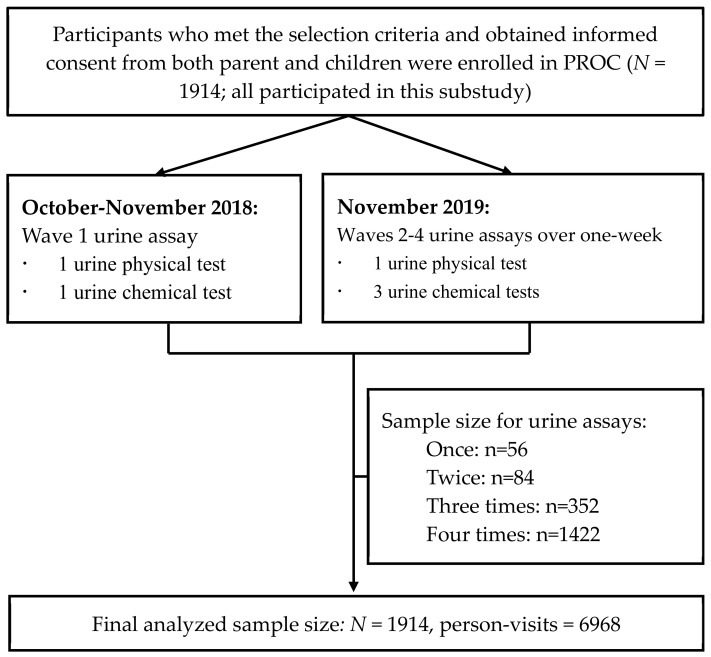
Flowchart of the procedure and frequency of urine collection for the study.

This study was nested within the PROC study conducted in Beijing enrolling 1914 children aged 6-8 years in six non-boarding primary schools (detailed elsewhere [18]). We conducted 4 waves of repeated urine assays for the same cohort. The single baseline urine was obtained in October–November 2018, and three subsequent urine assays were obtained from each child within a one-week span in November 2019 at the one-year follow-up visit. All 1914 participants provided at least one urine sample and were included in this study. Figure 1 illustrates the procedures and the number of children in each frequency category of urine collection.

### 2.2. Anthropometric Measurements

Anthropometric measurements were conducted by trained staff from October to November 2018 for the baseline survey and the anthropometric indicators in November 2019 were estimated using the measurements of the baseline and the following visit in September 2020. In short, the standing height was the average of two measurements and rounded to 0.1 cm. The weight of participants was measured in light clothes and rounded to 0.1 kg (detailed elsewhere [18]). Body mass index (BMI) was calculated as weight in kilograms divided by height in meter squared (kg/m^2^).

### 2.3. Blood Pressure Measurements

We performed three blood pressure measurements on the same day in June 2019 to estimate mean blood pressure by using an electronic sphygmomanometer (OMRON HBP-1300, Dalian, China). Systolic blood pressure (SBP) and diastolic blood pressure (DBP) were calculated as the average of the last two measurements.

### 2.4. Urine Collection and Measurements

Urine collection was conducted in four waves. The first wave was a baseline fasting urine assay in the morning in 2018. Wave 2 of 24 h urine samples were collected from Sunday morning to the following Monday morning, and waves 3–4 including two more fasting urine samples were collected on Wednesday and Friday morning within 1 week in November 2019. Urinalysis (including glucose [GLU], protein [PRO], bilirubin [BIL], urobilinogen [URO], pH, specific gravity [SG], blood [BLD], ketones [KET], nitrites [NIT], leukocytes [LEU], color tone, and turbidity) of wave 1 were performed using an automatic clinical chemistry analyzer Arkray AUTION MAX AX-4030 (Osaka, Japan) with AUTION Sticks. Urinalysis in wave 2 was performed by three instruments: URIT-500B (Guangxi, China), Dirui H-800 (Guangdong, China) and Combi Scan 500 (Lichtenfels, Germany), with corresponding urinalysis sticks to cope with many samples needing to be studied in a short time frame. Urine chemical tests (sodium, potassium, β_2_-MG and MA) of wave 1 and (sodium, potassium, creatinine, uric acid, β_2_-MG and MA) of waves 2–4 were performed using the automatic clinical chemistry analyzer Beckman Coulter AU5800 and AU680 (Osaka, Japan), respectively.

Based on spot urinary potassium excretion, we calculated the estimated 24 h urinary potassium excretion [19] and the estimated potassium intake [20]:

$$24\mathrm{hUK}=39.1\times 5.2\times {\left[\frac{0.1\times \mathrm{SpotUK}}{\mathrm{SpotUCr}}\left(-4.72\times \mathrm{Age}+7.58\times \mathrm{Weight}+6.09\times \mathrm{Height}-64.50\right)\right]}^{0.5}$$

24hKIntake=1.3×24hUK/1000 (g/d)

We defined the abnormal cutoffs value as <1.5 g/d [21] for insufficient potassium intake, >0.2 mg/L for elevated β_2_-MG and tubular damage [22] and ≥20 mg/L for elevated MA and glomerular damage [23].

### 2.5. Lifestyle Covariates and Definition

FVI, sleep duration, screen time, and physical activity were reported by parents. FVI was assessed via the 16-item Mediterranean Diet Quality Index in children and adolescents (KIDMED) [24] and grouped as sufficient (≥4/d) and insufficient (<4/d). Short sleep was defined as sleep duration <10 h/d; long screen time was defined as computer/cell phone screen time ≥2 h/d; insufficient physical activity was defined as <1 h/d.

### 2.6. Statistical Analysis

The main outcome indicators were the longitudinal urinary β_2_-MG and MA concentrations. Descriptive statistics are presented according to FVI status. Categorical variables are presented as counts and percentages. Continuous variables are described as the mean ± standard deviation (SD) or as median and interquartile range (IQR). Multiple imputations were performed for variables with missing values; thus, 50 complete datasets were obtained. Independent *t*-tests, the Mann–Whitney U test and the *χ*^2^ test were performed to compare the difference between sufficient and insufficient FVI groups. Linear mixed effects models were generated to determine the associations and coefficients with 95% confidence interval (95% CI) of renal damage indicators with FVI and urinary indicators, while the weekday and intra-wave of the urine assays were included as random effects. Sankey diagrams were used to visualize the associations of elevated β_2_-MG and MA with FVI status and potassium intake quartiles. The results in Table 1 are based on the first imputed dataset. We generated Table 2; Table 3 via valid statistical inferences of the parameters based on 50 datasets using PROC MIANALYZE. A two-tailed *p* value of 0.05 was used to determine statistical significance. All data were analyzed using Statistical Analysis System V.9.4 (SAS Institute Inc., Cary, NC, USA).

## 3. Results

### 3.1. Sociodemographic Characteristics

A total of 1914 children aged 6.6 ± 0.3 years old at the baseline were enrolled in this study. The prevalence of insufficient FVI is 48.6% (95% CI: 46.4%, 50.9%), and more common in boys (51.4%) than girls (45.9%). In the insufficient FVI group, short sleep and long screen time were significantly more prevalent, and urinary β_2_-MG and MA excretion of wave 1 were significantly higher than that in the sufficient FVI group. In wave 1–4, estimated 24 h urinary potassium excretion and estimated potassium intake were significantly lower in the insufficient FVI group. There were no significant differences in height, weight, BMI, SBP, DBP, prevalence of insufficient physical activity, and spot urinary potassium excretion between the two FVI groups (Table 1).

### 3.2. Binary Regression of Renal Damage Indicators, FVI, and Potassium Indicators

The unadjusted model 1 and adjusting for age, sex, and BMI model 2 suggested that estimated potassium intake was associated with insufficient FVI and urinary potassium excretion, urinary β_2_-MG and MA excretion were associated with insufficient FVI and estimated potassium intake, respectively. The coefficients of these associations remained stable and significant (all *p* ≤ 0.05) after adjusting for age, sex, BMI, SBP, sleep duration, screen time and physical activity (model 3). The results in model 3 showed that higher estimated potassium intake was negatively associated with insufficient FVI (*β* = −0.050, 95% CI: −0.072, −0.027), and positively associated with higher urinary potassium excretion (*β* = 0.013, 95% CI: 0.012, 0.014); higher urinary β_2_-MG excretion was positively associated with insufficient FVI (*β* = 0.012, 95% CI: 0.005, 0.020), and negatively associated with higher estimated potassium intake (*β* = −0.042, 95%CI: −0.052, −0.033); higher urinary MA excretion was positively associated with insufficient FVI (*β* = 0.717, 95% CI: 0.075, 1.359), and negatively associated with higher estimated potassium intake (*β* = −1.778, 95% CI: −2.600, −0.956) (Table 2).

### 3.3. Multivariable Regression of Renal Damage Indicators with Insufficient FVI and Insufficient Potassium Intake

The multivariable analysis (unadjusted model 1 and adjusting for age, sex, and BMI model 2) showed that urinary β_2_-MG and MA excretion increased with insufficient FVI and insufficient potassium intake. After adjusting for age, sex, BMI, SBP, sleep duration, screen time and physical activity (model 3), the urinary β_2_-MG excretion increased with insufficient FVI (*β* = 0.011, 95% CI: 0.004, 0.018) and insufficient potassium intake (*β* = 0.031, 95% CI: 0.023, 0.038); urinary MA excretion increased with insufficient FVI (*β* = 0.658, 95% CI: 0.017, 1.299) and insufficient potassium intake (*β* = 1.185, 95% CI: 0.492, 1.878) (Table 3).

### 3.4. Visualization of Early Renal Damage with Insufficient FVI and Quantiles of Potassium Intake

**Figure 2:**
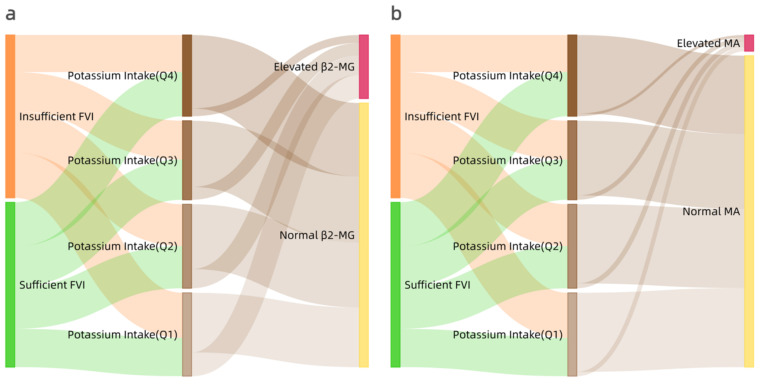
Sankey diagrams of renal damage with estimated potassium intake; elevated β_2_-MG (**a**) or elevated MA (**b**) urinary excretion were more likely to originate from lower quantile of potassium intake.

Tracking the proportion of children with elevated β_2_-MG (Figure 2a) or elevated MA (Figure 2b), they were more likely to appear in the lower quantile of estimated potassium intake. Lower potassium intake contributed a larger proportion of both indicators of renal damage.

## 4. Discussion

This longitudinal study with large sample size assessed the associations between FVI, estimated potassium intake and early renal damage among a general population of school-aged children in urban China. We found that children with insufficient FVI and low potassium intake were more likely to have increased urinary β_2_-MG and MA excretion, after controlling for potential sociodemographic and lifestyle confounders, including age, sex, BMI, SBP, sleep duration, screen time and physical activity. These findings highlight the potential importance of adequate FVI intake and potassium intake to prevent early renal damage in children.

The prevalence of insufficient FVI (<4/d) was 48.6% (95% CI: 46.4–50.9%), which was lower than previous surveys, i.e., 58.0% of urban Chinese children in 1982 and 72.7% in 2012 (both reported prevalence corresponds to the WHO recommendation of 80%, i.e., <4 servings/d instead of 5 servings/d) [6]. The difference in prevalence may be due to many factors, including economic status [25], time period differences, and methodologies. For instance, we used KIDMED surveys that focus on frequency of fruit and vegetable consumption each week rather than quantities of consumption. Even so, we could observe significant differences of estimated 24 h urinary potassium excretion and potassium intake in the insufficient FVI group. Furthermore, the longitudinal negative association we observed between estimated potassium intake and insufficient FVI validated such findings. We also found children with insufficient FVI are more likely to have short sleep [26] and long screen time [27]. The clustering of healthy lifestyle features suggests that a set of needed interventions should include increased FVI alongside shorter screen time and enough sleep to prevent early renal damage [27,28,29,30].

In both the unadjusted analysis and those that adjusted for age, sex, BMI, SBP, sleep duration, screen time, and physical activity, we observed consistent longitudinal positive associations between insufficient FVI (<4/d) and urinary β_2_-MG excretion, but not urinary MA excretion. This finding suggests that insufficient FVI leads to earlier renal tubular stress than glomerular stress, and evidence suggests tubular damage precede glomerular damage [31]. Clinical management practices in CKD patients with glomerular or tubular abnormalities include dietary intervention with base-producing foods such as fruits and vegetables to increase potassium absorption [9] and reduce metabolic acidosis [32]. We observed a longitudinal negative association between urinary β_2_-MG or MA excretion and estimated potassium intake. Many studies have evaluated the effect of potassium intake on renal damage, and most results reached a consensus that insufficient potassium intake was associated with an increased risk of CKD in different populations [11,16,17], while our result provided the same evidence in the general pediatric population.

We found urinary β_2_-MG and MA excretion increased with insufficient FVI (<4/d) and insufficient potassium intake (<1.5 g/d) in multivariable models, adjusting for age, sex, BMI, SBP, sleep duration, screen time, and physical activity. This finding suggests a consistent synergistic effect of FVI and potassium intake on early renal damage, i.e., at least 4/d FVI and 1.5 g/d potassium intake may mitigate or prevent glomerular or tubular damage. Our data support a conclusion that school-aged children should have no less than 4 kinds of FVI daily, even independent of the quantity of fruit and vegetable consumption. Moreover, the Sankey diagrams show that children with renal tubular or glomerular damage more likely originate from the lower quantile of the potassium intake group. The comprehensive analysis of the association between FVI, potassium intake and early renal damage leads to the robust conclusion that insufficient FVI and low potassium intake are associated with early renal damage and have a synergistic effect.

The major strength of this study was its use of multiple measures and longitudinal data from a population-based healthy children cohort in China. The imputation method for estimating missing data can maximize the use of information and avoid reporting bias. We selected appropriate linear mixed effects models and covariates to adjust for associations between renal outcomes and independent variables, especially SBP and lifestyle factors such as sleep duration, screen time, and physical activity. Therefore, our conclusions that insufficient FVI and low potassium intake aggravate early renal damage can be placed into a more relevant public health context. This study was limited by not considering other renal function indicators. Urine β_2_-MG, MA, and potassium were tested via different machines due to the limited capacity of each testing site, but these effects were minimized by longitudinal data.

## 5. Conclusions

In conclusion, we present the longitudinal effects of FVI and potassium intake on early renal damage in school-aged children in China. We found that insufficient FVI and low potassium intake aggravate early renal damage in children. Our findings underscore the necessity of advocating more FVI for children to protect renal function from early damage in the context of other healthy lifestyle choices such as enough sleep, less screen time, and more physical activity.

## Figures and Tables

**Table 1:** Descriptive characteristics of 6–9 year old children categorized by sufficiency of fruit and vegetable intake or not in Beijing, China (*N* = 1914).

Factors	Total	Sufficient FVI (≥4/d)	Insufficient FVI (<4/d)	*p*
Sex ^1^				0.018
Boy [n (%)]	956 (50.0)	465 (47.3)	491 (52.7)	
Girl [n (%)]	958 (50.0)	518 (52.7)	440 (47.3)	
Age (year) ^2^	6.6 ± 0.3	6.6 ± 0.3	6.6 ± 0.3	0.09
Height (cm) ^2^	122.5 ± 5.3	122.3 ± 5.4	122.6 ± 5.3	0.25
Weight (kg) ^2^	24.8 ± 5.9	24.7 ± 5.9	24.8 ± 5.9	0.72
BMI (kg/m^2^) ^2^	16.4 ± 2.9	16.4 ± 2.9	16.4 ± 2.9	0.94
SBP (mmHg) ^2^	101 ± 8	101 ± 8	101 ± 9	0.27
DBP (mmHg) ^2^	56 ± 6	56 ± 6	56 ± 6	0.93
Short sleep (<10 h/d) ^1^	1441 (75.3)	706 (71.8)	735 (78.9)	<0.001
Long screen time (≥2 h/d) ^1^	95 (5.0)	37 (3.8)	58 (6.2)	0.013
Insufficient physical activity (<1 h/d) ^1^	1451 (75.8)	739 (75.2)	712 (76.5)	0.51
Spot urinary potassium excretion (mmol/L) ^2^				
Wave 1	27.36 ± 12.53	27.41 ± 12.54	27.30 ± 12.53	0.85
Wave 2	30.42 ± 12.30	30.34 ± 12.47	30.50 ± 12.12	0.77
Wave 3	26.59 ± 12.86	26.82 ± 13.00	26.36 ± 12.74	0.45
Wave 4	28.00 ± 13.56	28.26 ± 13.45	27.73 ± 13.68	0.46
Estimated 24 h urinary potassium excretion (mg/d) ^2^				
Wave 1	1253.5 ± 370.3	1272.4 ± 371.1	1233.6 ± 368.7	0.022
Wave 2	1363.1 ± 260.2	1383.4 ± 267.0	1342.4 ± 251.5	<0.001
Wave 3	1116.7 ± 276.5	1134.8 ± 270.8	1097.9 ± 281.3	0.005
Wave 4	1114.4 ± 266.6	1131.0 ± 263.3	1097.3 ± 269.1	0.017
Estimated 24 h potassium intake (g/d) ^2^				
Wave 1	1.63 ± 0.48	1.65 ± 0.48	1.60 ± 0.48	0.022
Wave 2	1.77 ± 0.34	1.80 ± 0.35	1.75 ± 0.33	<0.001
Wave 3	1.45 ± 0.36	1.48 ± 0.35	1.43 ± 0.37	0.005
Wave 4	1.45 ± 0.35	1.47 ± 0.34	1.43 ± 0.35	0.016
Spot urinary β_2_-MG excretion (mg/L) ^3^				
Wave 1	0.08 (0.04–0.13)	0.07 (0.04–0.12)	0.08 (0.05–0.13)	0.011
Wave 2	0.15 (0.12–0.18)	0.14 (0.12–0.18)	0.15 (0.12–0.19)	0.066
Wave 3	0.16 (0.13–0.21)	0.16 (0.13–0.20)	0.16 (0.13–0.21)	0.19
Wave 4	0.16 (0.13–0.21)	0.16 (0.13–0.21)	0.16 (0.13–0.21)	0.52
Spot urinary MA excretion (mg/L) ^3^				
Wave 1	9.13 (6.45–12.56)	8.85 (6.36–12.18)	9.28 (6.53–12.96)	0.019
Wave 2	6.70 (6.10–8.50)	6.60 (6.10–8.30)	6.70 (6.10–8.90)	0.027
Wave 3	7.00 (6.20–9.10)	6.90 (6.20–9.00)	7.00 (6.20–9.40)	0.35
Wave 4	6.80 (6.00–8.90)	6.70 (6.00–8.80)	6.85 (6.00–9.10)	0.16

(FVI: fruit and vegetable intake, BMI: body mass index, SBP: systolic blood pressure, DBP: diastolic blood pressure, β_2_-MG: β_2_-microglobulin, MA: microalbumin.). ^1^ Comparison by FVI status using χ^2^ test. ^2^ Mean and standard deviation (SD) compared by FVI status using independent *t*-test. ^3^ Median and interquartile ranges (IQR) compared by FVI status using the Mann–Whitney U test.

**Table 2:** Bivariate associations of renal damage indicators, FVI and potassium indicators using linear mixed effects models among 6–9 year old children, Beijing, China.

Dependent Variable	Independent Variable	Model 1		Model 2		Model 3	
		Estimate (95%CI)	*p*	Estimate (95%CI)	*p*	Estimate (95%CI)	*p*
Estimated pota-ssium intake	Insufficient FVI	−0.049 (−0.071, −0.027)	<0.001	−0.049 (−0.071, −0.027)	<0.001	−0.050 (−0.072, −0.027)	<0.001
	Spot urinary potassium	0.013 (0.012, 0.014)	<0.001	0.013 (0.012, 0.014)	<0.001	0.013 (0.012, 0.014)	<0.001
β_2_-MG	Insufficient FVI	0.011 (0.004, 0.018)	0.003	0.011 (0.004, 0.018)	0.004	0.012 (0.005, 0.020)	<0.001
	Estimated potassium intake	−0.043 (−0.052, −0.034)	<0.001	−0.042 (−0.052, −0.033)	<0.001	−0.042 (−0.052, −0.033)	<0.001
MA	Insufficient FVI	0.616 (−0.020, 1.253)	0.058	0.669 (0.034, 1.304)	0.039	0.717 (0.075, 1.359)	0.029
	Estimated potassium intake	−1.837 (−2.656, −1.019)	<0.001	−1.753 (−2.574, −0.933)	<0.001	−1.778 (−2.600, −0.956)	<0.001

Model 1: unadjusted; Model 2: adjusting for age, sex, BMI; Model 3: adjusting for age, sex, BMI, SBP, sleep duration, screen time and physical activity. (FVI: fruit and vegetable intake, BMI: body mass index, SBP: systolic blood pressure, β_2_-MG: β_2_-microglobulin, MA: microalbumin. All models included two random effects: the weekday and wave of the urine assay.).

**Table 3:** Multivariable associations of renal damage indicators with insufficient FVI and insufficient potassium intake using linear mixed effects models among 6–9 year old children, Beijing, China.

Dependent Variable	Independent Variable	Model 1		Model 2		Model 3	
		Estimate (95%CI)	*p*	Estimate (95%CI)	*p*	Estimate (95%CI)	*p*
β_2_-MG	Intercept	0.142 (0.104, 0.180)	<0.001	0.090 (−0.004, 0.184)	0.061	0.043 (−0.058, 0.145)	0.40
	Insufficient FVI	0.009 (0.002, 0.017)	0.010	0.009 (0.002, 0.017)	0.011	0.011 (0.004, 0.018)	0.003
	Insufficient potassium intake	0.031 (0.023, 0.039)	<0.001	0.031 (0.023, 0.038)	<0.001	0.031 (0.023, 0.038)	<0.001
MA	Intercept	8.940 (7.911, 9.969)	<0.001	6.614 (−0.662, 13.890)	0.075	2.228 (−5.736, 10.193)	0.58
	Insufficient FVI	0.558 (−0.077, 1.193)	0.085	0.612 (−0.022, 1.246)	0.059	0.658 (0.017, 1.299)	0.044
	Insufficient potassium intake	1.255 (0.563, 1.947)	<0.001	1.171 (0.479, 1.863)	<0.001	1.185 (0.492, 1.878)	<0.001

Model 1: unadjusted; Model 2: adjusting for age, sex, BMI; Model 3: adjusting for age, sex, BMI, SBP, sleep duration, screen time and physical activity. FVI: fruit and vegetable intake, BMI: body mass index, SBP: systolic blood pressure, β_2_-MG: β_2_-microglobulin, MA: microalbumin. All models included two random effects: the weekday and wave of the urine assay.

## Data Availability

The data that support the findings of this study are not publicly available but are available from the corresponding author on reasonable request.
